# Integrin α10β1-selected mesenchymal stem cells reduced hypercoagulopathy in a porcine model of acute respiratory distress syndrome

**DOI:** 10.1186/s12931-023-02459-6

**Published:** 2023-05-31

**Authors:** Dag Edström, Anna Niroomand, Martin Stenlo, Kristina Uvebrant, Deniz A. Bölükbas, Gabriel Hirdman, Ellen Broberg, Hooi Ching Lim, Snejana Hyllén, Evy Lundgren-Åkerlund, Leif Pierre, Franziska Olm, Sandra Lindstedt

**Affiliations:** 1 Department of Cardiothoracic Anaesthesia and Intensive Care, Lund University Hospital, Lund, Sweden; 2 Wallenberg Center for Molecular Medicine, Lund University, Lund, Sweden; 3 Lund Stem Cell Center, Lund University, Lund, Sweden; 4 Department of Clinical Sciences, Lund University, Lund, Sweden; 5 Rutgers Robert University, New Brunswick, NJ USA; 6 Xintela AB, Lund, Sweden; 7 Department of Experimental Medical Sciences, Lung Bioengineering and Regeneration, Lund University, Lund, Sweden; 8 Department of Cardiothoracic Surgery and Transplantation, Lund University Hospital, 22242 Lund, Sweden

**Keywords:** Acute respiratory distress syndrome, Integrin α10β1 mesenchymal stem cells, Lung disease, Advanced therapeutic medicinal products

## Abstract

Mesenchymal stem cells (MSCs) have been studied for their potential benefits in treating acute respiratory distress syndrome (ARDS) and have reported mild effects when trialed within human clinical trials. MSCs have been investigated in preclinical models with efficacy when administered at the time of lung injury. Human integrin α10β1-selected adipose tissue-derived MSCs (integrin α10β1-MSCs) have shown immunomodulatory and regenerative effects in various disease models. We hypothesized that integrin α10β1 selected-MSCs can be used to treat a sepsis-induced ARDS in a porcine model when administering cells after established injury rather than simultaneously. This was hypothesized to reflect a clinical picture of treatment with MSCs in human ARDS. 12 pigs were randomized to the treated or placebo-controlled group prior to the induction of mild to moderate ARDS via lipopolysaccharide administration. The treated group received 5 × 10^6^ cells/kg integrin α10β1-selected MSCs and both groups were followed for 12 h. ARDS was confirmed with blood gases and retrospectively with histological changes. After intervention, the treated group showed decreased need for inotropic support, fewer signs of histopathological lung injury including less alveolar wall thickening and reduction of the hypercoagulative disease state. The MSC treatment was not associated with adverse events over the monitoring period. This provides new opportunities to investigate integrin α10β1-selected MSCs as a treatment for a disease which does not yet have any definitive therapeutic options.

**Supplementary Information:**

The online version contains supplementary material available at 10.1186/s12931-023-02459-6.

## Introduction

Ten percent of intensive care unit admissions globally fulfill criteria for acute respiratory distress syndrome (ARDS), making the burden of ARDS on mortality and morbidity significant [1]. The recent COVID-19 pandemic has only exacerbated these figures, causing a worldwide increase in disease frequency. ARDS is characterized by respiratory failure resulting from excessive alveolocapillary permeability precipitating pulmonary oedema [2]. The pulmonary dysfunction that results is assessed with the arterial oxygenation saturation relative to the fraction of inspired air (PaO_2_/FiO_2_ ratio) and ARDS is defined by a ratio below 300 mmHg [3]. The onset of ARDS is associated with mortality rates of approximately 35% to 45% of patients [1]. Standard management currently includes prone positioning and lung-protective ventilation [4], however, there has been limited success in developing pharmaceutical interventions. There are no existing treatments which have been shown to be consistently effective [2].

The lack of promising and effective therapies has driven interest in the use of advanced therapeutic medicinal products. Of these, mesenchymal stem cells (MSCs) and their secreted factors have been effective in preclinical studies. MSCs originate from a variety of tissues, including bone marrow and adipose tissue and are known to participate in both tissue repair and immunomodulation. The administration of MSCs in lung injury and other diseases has correlated with decreased levels of inflammatory cytokines and increased levels of anti-inflammatory IL-10 [5–7]. In models of severe lung injury, MSC treatment has led to improved oxygenation and reduced lung edema [8]. In *E. coli-*damaged human lungs placed on ex vivo lung perfusion, MSCs given endotracheally can lead to reduced permeability to protein and increased fluid clearance in a preclinical setting [9, 10]. This current study explores MSCs within a lung injury model but is novel for its administration of cells only following injury confirmed according to the Berlin criteria of oxygenation. In this case, a clinical definition of lung injury was established by two consecutive blood gases fifteen minutes apart which fell within the Berlin criteria without any signs of cardiac failure and a positive end expiratory pressure of at least 5 cmH_2_O. This entailed the administration of the treatment only after confirmation of injury. MSCs have also been trialled for clinical use in humans with established ARDS, with the phase 2a START trial establishing safety and a lack of adverse effects at a dose of 10 × 10^6^ cells per kg predicted body weight without reported treatment effects on oxygenation and survival [11]. Allogeneic MSCs are a promising therapeutic intervention but treatment effect could be related to timing of administration, as hypothesized in this study. MSCs are promising given their low immunogenicity, in which an absence of co-stimulator molecules helps the cells avoid elimination by the immune system. Yet, MSC preparations may display substantial heterogeneity of cell types that varies between donors and between tissue sources, which has led to varying results across studies [12]. Selection of MSCs to generate homogenous MSC preparations thus has the potential to improve the therapeutic outcome of the treatment [13]. Previous studies have suggested a functional advantage by selecting the MSCs using the collagen binding cell surface receptor integrin α10β1 which also leads to a consistent product [14, 15]. Since integrin α10β1-MSCs derived from adipose tissue (integrin α10β1-MSCs) have been shown to exert effective immunomodulatory activity and tissue repair capacity, we investigated a potential treatment effect in a porcine model of ARDS [14, 15]. ARDS was induced via administration of lipopolysaccharide (LPS) derived from *E. coli.* Previous studies have given MSCs simultaneously with injury onset. Hypothesizing that treatment timing may relate to efficacy, the MSCs in this study were administered after confirmed ARDS. This current work then investigated the administration of integrin α10β1-MSCs as a potential treatment in a sepsis-like porcine ARDS model to ameliorate the histological signs of damage and improve hypercoagulation.

## Materials and methods

### Cells isolation, selection, and characterization

Human integrin α10β1-selected MSCs were used in this study suspended in cryopreservation medium at a concentration of 5 × 10^6^ cells/mL (XSTEM®, Xintela AB, Lund, Sweden). Human adipose tissue-derived mesenchymal stem cells were isolated from lipoaspirates of healthy donors who provided informed consent under the Xintela Tissue Establishment license. The stromal vascular fraction (SVF) was isolated and seeded according to methods in Zuk et al. [16]. Briefly, lipoaspirate samples were enzymatically digested and washed. The resultant SVF was seeded in MSC expansion medium (Miltenyi Biotech, Bergisch Gladbach, Germany) in cell culture flasks. When cells reached ~ 80% confluence, the adherent cells were harvested and expanded. After 3 passages, those cells with high expression of integrin α10β1 were isolated using an integrin α10β1 antibody (Xintela AB, Lund Sweden) and magnetic beads (Miltenyi Biotec, Bergisch Gladbach, Germany). The integrin α10β1-selected cells were further expanded prior to cryopreservation at a concentration of 5 × 10^6^ cells/mL of cryopreservation medium and stored in liquid nitrogen [14].

The integrin α10β1-selected cells fulfilled the International Society for Cell & Gene Therapy (ISCT) criteria for MSC identity: plastic adherent, ≥ 95% cell surface expression of CD73, CD90 and CD105; ≤ 2% cell surface expression of the hematopoietic markers CD34, CD45, CD19, CD11b and HLA-DR; and trilineage differentiation capacity (osteoblasts, adipocytes and chondrocytes) [14, 17]. All the cell culturing procedures were in accordance with good manufacturing practices (GMP).

### Animal preparation

The animals were randomized prior to the start of the study to either the treatment group or the placebo group.

Placebo—the placebo group refers to those animals randomized to receive a placebo of cryopreservation medium after the establishment of ARDS. A sepsis-like condition was induced with LPS, after which this group received the placebo medium (n = 6).

Treated—the treated group refers to those animals randomized to receive MSC treatment after the establishment of ARDS. A sepsis-like condition was induced with LPS, after which this group received 5 × 10^6^ cells/kg (n = 6).

**Fig. 1:**
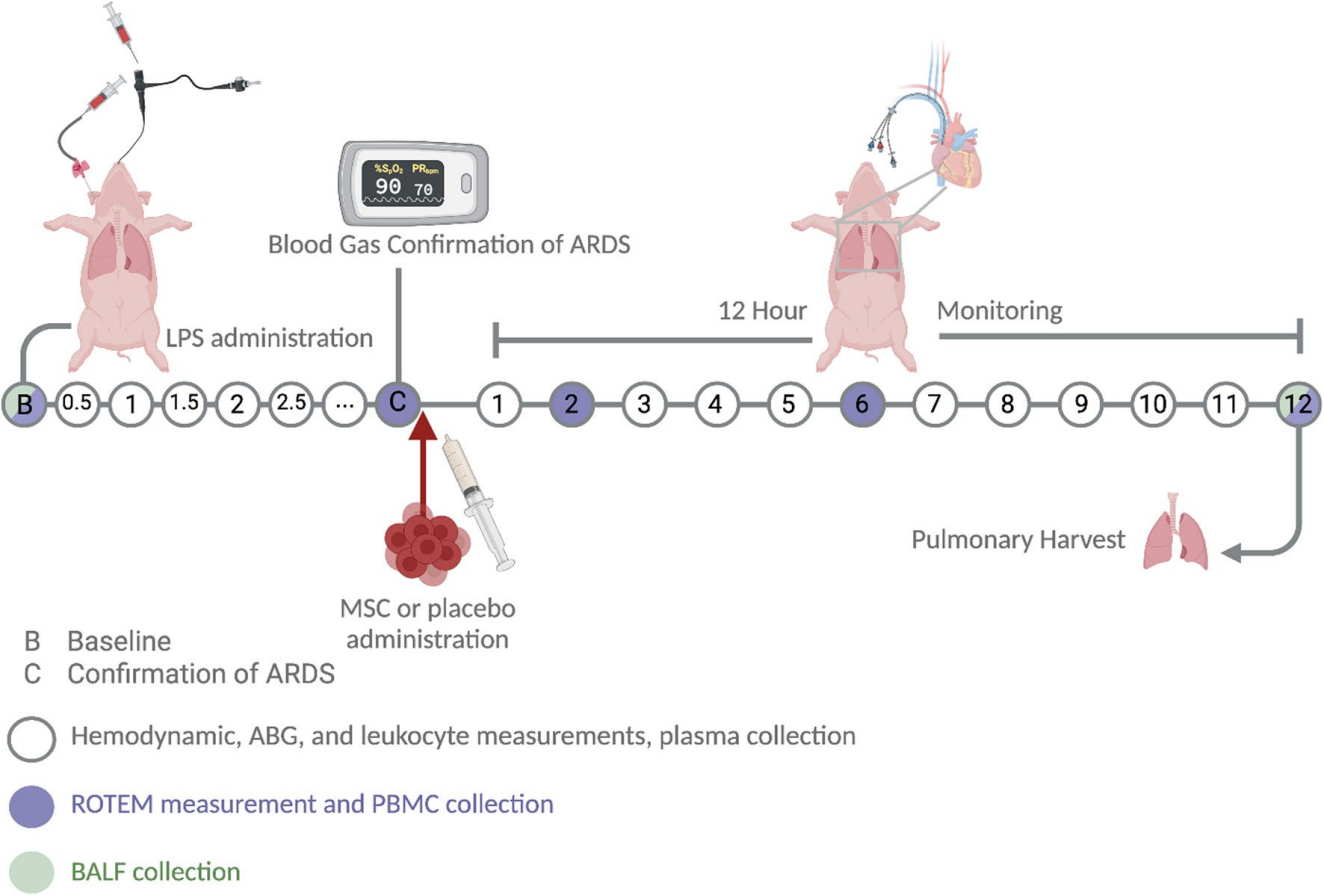
Overview of in vivo experimental set-up and in vitro follow-up. Sepsis-like acute respiratory distress syndrome was induced through a dual-hit administration of lipopolysaccharide (LPS), intravenously for a systemic response and endotracheally through a bronchoscope for an immediate local effect in the lungs. The ARDS lung injury was confirmed with two consecutive blood gases. Thereafter, pigs were randomized to either placebo or MSC treatment, where 5 × 10^6^ cells/kg were administered. The pigs were closely monitored for up to 12 h, which was the defined endpoint of the experiment. Hemodynamic measurements, vital measurements, pulse oximetry, arterial blood gases, leukocyte levels and coagulation parameters were measured, and peripheral blood mononuclear cells were isolated at defined timepoints. Bronchoalveolar lavage fluid (BALF) was collected at the beginning and at the endpoint of the experiment. Finally, lungs were harvested and tissue prepared for histological analysis and the 5-day in vitro continuation of the experiment through precision cut lung slices. Created with Biorender.com

A total of 12 pigs with a mean weight of 40 kg were premedicated with xylazine (Rompun® vet. 20 mg/mL; Bayer AG, Leverkusen, Germany; 2 mg/kg) and ketamine (Ketaminol® vet. 100 mg/mL; Farmaceutici Gellini S.p.A., Aprilia, Italy; 20 mg/kg). A peripheral intravenous (IV) line was inserted in the earlobe. General anaesthesia was accomplished with ketamine (Ketaminol® vet), midazolam (Midazolam Panpharma®, Oslo, Norway) and fentanyl (Leptanal®, Lilly, France) infusions. Mechanical ventilation was established using a Siemens-Elema ventilator (Servo 900C, Siemens, Solna, Sweden). The animals were intubated with a 7.5 size endotracheal tube and then a surgical tracheostomy was performed with a 7.5 endotracheal tube. The ventilator was set to volume-controlled ventilation (VCV) with the flow pattern switch in “constant flow” which lowers the peak pressures according to the manufacturer’s instructions. Inspiration time was set to 25% with pause time 10% to give an I:E ratio of 1:2. Ventilation was adjusted to maintain carbon dioxide levels (PaCO_2_) between 33 and 41 mmHg. Tidal volume (Vt) was kept at 6–8 mL/kg. Dynamic compliance was calculated by the equation ${C}_{dyn}= \frac{{V}_{T}}{(peak\;pressure-PEEP)}$. A pulmonary artery catheter (Swan-Ganz CCOmbo V and Introflex, Edwards Lifesciences Services GmbH, Unterschleissheim, Germany) was inserted in the right internal jugular vein and an arterial line (Secalon-T™, Merit Medical Ireland Ltd, Galway, Ireland) was placed in the right common carotid artery. A urinary catheter was surgically inserted in the bladder. A rectal probe was used to monitor temperature continuously. Figure 1 shows the overview of the experimental set up.

### Ventilatory settings and measurements before lung injury induction

Baseline measurements included 30 min of standard ventilation with volume-controlled ventilation (VCV), a tidal volume (Vt) of 6–8 mL/kg, a peak end-expiratory pressure (PEEP) of 5, an inspiratory-expiratory ratio (I:E) of 1:2, a respiratory rate (RR) of 20–25 breaths/min, and a fraction of inspired oxygen (FiO_2_) of 0.5.

### Induction of ARDS using lipopolysaccharide

Lipopolysaccharide (LPS) from gram-negative bacteria *Escherichia coli* (O111:B4, Sigma-Aldrich, Merck KGaA, Darmstadt, Germany) was used to induce an ARDS, as previously described [18].

LPS was diluted in two different solutions, with one dose for endotracheal (ET) instillation (0.33 mg/kg) and one intravenous infusion (0.5 µg/kg/min). The animals were given one dose ET distributed equally between the left and right upper and lower lobes by bronchoscopy. This was followed by an intravenous infusion (0.5 µg/kg/min) for one hour. After one hour, the infusion rate was reduced by 50% for another hour. All LPS-treated animals developed a septic-like condition with hemodynamic instability and required continuous infusion of norepinephrine (40 µg/mL, 0.05–2 µg/kg/min) (Pfizer AB, Sollentuna, Sweden) and dobutamine (2 mg/mL, 2.5–5 µg/kg/min) (Hameln Pharma Plus GmbH, Hameln, Germany). Fluid loss was compensated with Ringer’s acetate (Baxter Medical AB, Kista, Sweden) in all animals. Albumin was given for volume expansion based on a mean arterial pressure (MAP) below 65 mmHg and decreased urinary output below 1 mL/kg/h.

The different ARDS stages were defined according to the PaO_2_/FiO_2_ ratio. Mild ARDS was defined as a ratio between 201 and 300 mmHg, moderate ARDS between 101 and 200 mmHg, and severe ARDS as ≤ 100 mmHg. Animals were confirmed as having ARDS following two separate arterial blood gases falling below 300 mmHg within a 15-min interval.

After confirmed ARDS the animals were intravenously given either 5 × 10^6^ cells/kg or the equivalent volume of freezing medium as placebo and followed over 12 h.

### Arterial blood gases

Arterial blood gases were analysed every 30 min with an ABL 90 FLEX blood gas analyser (Radiometer Medical ApS, Brønshøj, Denmark), and normalized to the blood temperature of 37 °C according to clinical standards.

### Hemodynamic monitoring and vital measurements

Hemodynamic parameters, vitals and pulse oximetry were measured continuously and recorded every 30 min using thermodilution with a Swan-Ganz catheter and an arterial line. Heart rate (HR), percutaneous oxygen saturation (SpO_2_), systolic blood pressure (SBP), diastolic blood pressure (DBP), mean arterial pressure (MAP), central venous pressure (CVP), cardiac output (CO), systolic pulmonary pressure (SPP), diastolic pulmonary pressure (DPP), mean pulmonary pressure (MPP), pulmonary artery wedge pressure (PAWP), systemic vascular resistance (SVR), and pulmonary vascular resistance (PVR) were recorded.

### Measurements of cytokine levels in plasma and bronchoalveolar lavage fluid (BALF)

Measurements of cytokine levels in the plasma were taken at baseline, and every 60 min. Bronchoalveolar lavage fluid (BALF) was taken at baseline and at the end of the experiment. IL-1β, IL-4, IL-6, IL-8, IL-10, IL-12p40, IFN-γ and TNF-α levels were evaluated using xMAP technology with a Luminex 200 system according to manufacturer’s instructions (n = 12). IFN-α levels (n = 9) were analysed with the multiplex kit Cytokine & Chemokine 9-Plex Porcine ProcartaPlex™ Panel 1 (Thermo Fisher Scientific Cat. No. EPX090-60829-901, Waltham, MA, USA) according to the manufacturer’s instructions, using a Bioplex-200 system (BioRad, Hercules, CA, USA).

### Blood cell counts

Leukocyte, neutrophils, and total white blood cell counts were measured in EDTA anti-coagulated whole blood samples using a Sysmex KX-21N automated haematology analyser (Sysmex, Milton Keynes, UK) every 30 min in the induction phase and every 60 min post-treatment or placebo injection.

### Rotational thromboelastometry (ROTEM)

Measurements of haemostasis in whole blood were taken at baseline anticoagulated with sodium citrate tubes (BD Biosciences New Jersey, USA), before treatment/placebo administration, at 2 h and 6 h post-injection, and at the endpoint of the experiment. As each sample required substantial measurement time within the ROTEM machine, the timing of each sample was stratified post-operatively into early-, mid-, and late-post-treatment groups. The blood samples were analysed using a ROTEM sigma (Tem Innovations GmbH, Munich, Germany) to measure the extrinsic pathway (EXTEM), the intrinsic pathway (INTEM) and heparin modifications (HEPTEM).

### Histopathological analyses

Biopsies were taken from the left lung from the upper and lower regions at the end of the experiment. Biopsies were taken at the same location in all animals (Fig. 5b). Biopsies were fixed in 10% neutral buffered formalin solution (Sigma Aldrich, Darmstadt, Germany) at 4 °C overnight. Formalin-fixed tissues were subjected to a graded ethanol series and isopropanol (both Fisher Scientific, UK) prior to paraffin embedding. 4 μm thick sections were cut and collected on SuperFrost plus microscope slides. Sections were de-paraffinized, starting with Xylen (100%, Histolab, Askim, Sweden) followed by immersion in a graded ethanol series (from 99.99 to 70%) ended with rinses in distilled water. Sections were then stained with haematoxylin and eosin (H&E, Histolab), and were then dehydrated in ethanol, 96% and 99.99%, ending with xylene (100%). Sections were mounted in Pertex (Histolab, Sweden).

Slides from each animal, with at least 19 individual tissue sections from the upper or lower lobes, were scored for lung injury by a blinded pathologist with experience in porcine lung injury models. Findings were documented concerning alveolar wall thickening, and presence of fibrosis, haemorrhage, capillary congestion, alveolar macrophages, acute inflammation, or chronic inflammation. The total amount of pathological findings were computed as 0 to 3 points for degree of alveolar wall thickening (distribution and maximum density) and given 1 point for any other pathological finding listed above.

Representative bright-field images were acquired with a Nikon Eclipse Ts2R microscope (Nikon, Japan), of the left upper and lower lung regions (Fig. 6b.)

### Peripheral blood mononuclear cell isolation and flow cytometric analysis

Peripheral blood mononuclear cells (PBMCs) were isolated from heparinized whole blood collected at different time points (BD Vacutainer® Heparin Tubes, BD Biosciences, New Jersey, US) using Lymphoprep tubes for density gradient centrifugation (Alere Technologies AS, Oslo, Norway). The PBMCs were cryopreserved and stored in liquid nitrogen until further analysis. The PBMCs were thawed and washed with cell staining buffer (BioLegend, San Diego, USA). After blocking, the cells were stained with monoclonal antibodies against CD3-FITC (clone PPT3), CD4-Alexa 647 (clone b38c6c7), CD8-PE (clone 11/295/33), CD14-FITC (clone MIL2), CD163-PE (clone 2A10/11), CD21-Alexa 647 (clone CC51), CD45-Alexa 647 (clone K252.1E4) or SLA-DR-FITC (clone 2E9/13). All antibodies were purchased from Bio-Rad. Cells were acquired using a CytoFLEX flow cytometer (Beckman Coulter, Indianapolis, IN, US). Dead cells were excluded by 7-amino-actinomycin (7-AAD, BioLegend) staining, and doublets were excluded by gating on FSC-H vs FSC-A (Additional file 1: Fig. S1). Data was analysed using FlowJo software version 10 (BD Bioscience, Franklin Lakes, New Jersey, USA).

### Wet dry-weight ratio

Pulmonary oedema was examined by measuring the wet weight to dry weight ratio in lung tissue from the lower lobe at the end of the experiment. Proximal lung tissue pieces harvested were weighed, lyophilized for 24 h, and then weighed again. The ratio between the wet and dry weight was then calculated.

### Immune cell counts within BALF

BALF collected from the baseline and experimental end-point were counted with a NucleoCounter NC-3000 (Chemometec, Allerød, Hovedstaden, Denmark) using the viability and cell count assay and the fluid was then diluted to 10 × 10^5^ cells/mL in phosphate buffered saline (HyClone, GE Healthcare Life Sciences, Logan, Utah, USA). 100 µL were spun onto slides using a CytoSpin 3 (Shandon CytoSpin cytocentrifuge, Thermo Shandon, Chesire, UK). Slides were stained with May Grunewald Giemsa stain (Giemsa stain from Sigma-Aldrich GS500-500ML, May-Grunwald stain from Sigma-Aldrich MG500-500ML, Darmstadt, Germany), and between 200 and 300 cells were counted manually by three independent blinded researchers to differentiate between mononuclear and multinuclear cells.

### Precision cut lung slices (PCLS)

At the end of the experiment, four lungs from each group were explanted en bloc and flushed with sterile normal saline solution. These lungs were then kept at 4 °C until the PCLS were generated, which was done through filling an airway with 3% agarose (Sigma-Aldrich) by weight and slicing using a vibratome (7000SMZ-2 Vibratome, Campden Instruments, Loughborough, England, see Additional file 1: Methods for greater detail). Punches were maintained in culture for 24, 48, and 120 h. At the appropriate time point, punches were fixed in 10% formalin (Fisher Scientific, UK) and underwent processing, paraffin-embedding, and haematoxylin and eosin staining as reported for the biopsies. Bright-field images were acquired on an Olympus CKX53 microscope (Olympus Life Sciences, Tokyo, Japan) and then scored by three blinded scorers under a modified version of the biopsy scoring criteria.

### Statistics

Continuous variables were reported as mean and standard deviation (SD). Statistically significant differences between groups were tested with the Student’s T-test when comparing two groups and within groups with ANOVA when data were normally distributed. Most analyses were conducted with the Mann–Whitney test and the Kruskal–Wallis tests as data were not normally distributed. A Chi-Squared test was performed to analyse observed frequencies of categorical variables. The Kaplan–Meier curve was plotted with GraphPad Prism and groups were compared with the log-rank test. All graphs and statistical analyses were generated using GraphPad Prism 9.1. Statistical significance was defined as: p < 0.001 (***), p < 0.01 (**), p < 0.05 (*), and p > 0.05 (not significant, ns).

### Study approval

The study was approved by the local Ethics Committee for Animal Research (Dnr 5.2.18-4903/16, and Dnr 5.2.18-8927/16). All animals received care according to the USA Principles of Laboratory Animal Care of the National Society for Medical Research, Guide for the Care and Use of Laboratory Animals, National Academies Press (1996).

## Results

### ARDS state was established by LPS administration

The animals were randomized prior to the start of the study to either the treatment group or the placebo group and an overview of the experimental procedure is shown in Fig. 1.

Placebo—the placebo group refers to those animals randomized to receive a placebo of cryopreservation medium after the establishment of ARDS. A sepsis-like condition was induced with LPS, after which this group received the placebo medium (n = 6).

Treated—the treated group refers to those animals randomized to receive MSC treatment after the establishment of ARDS. A sepsis-like condition was induced with LPS, after which this group received 5 × 10^6^ cells/kg (n = 6).

**Fig. 2:**
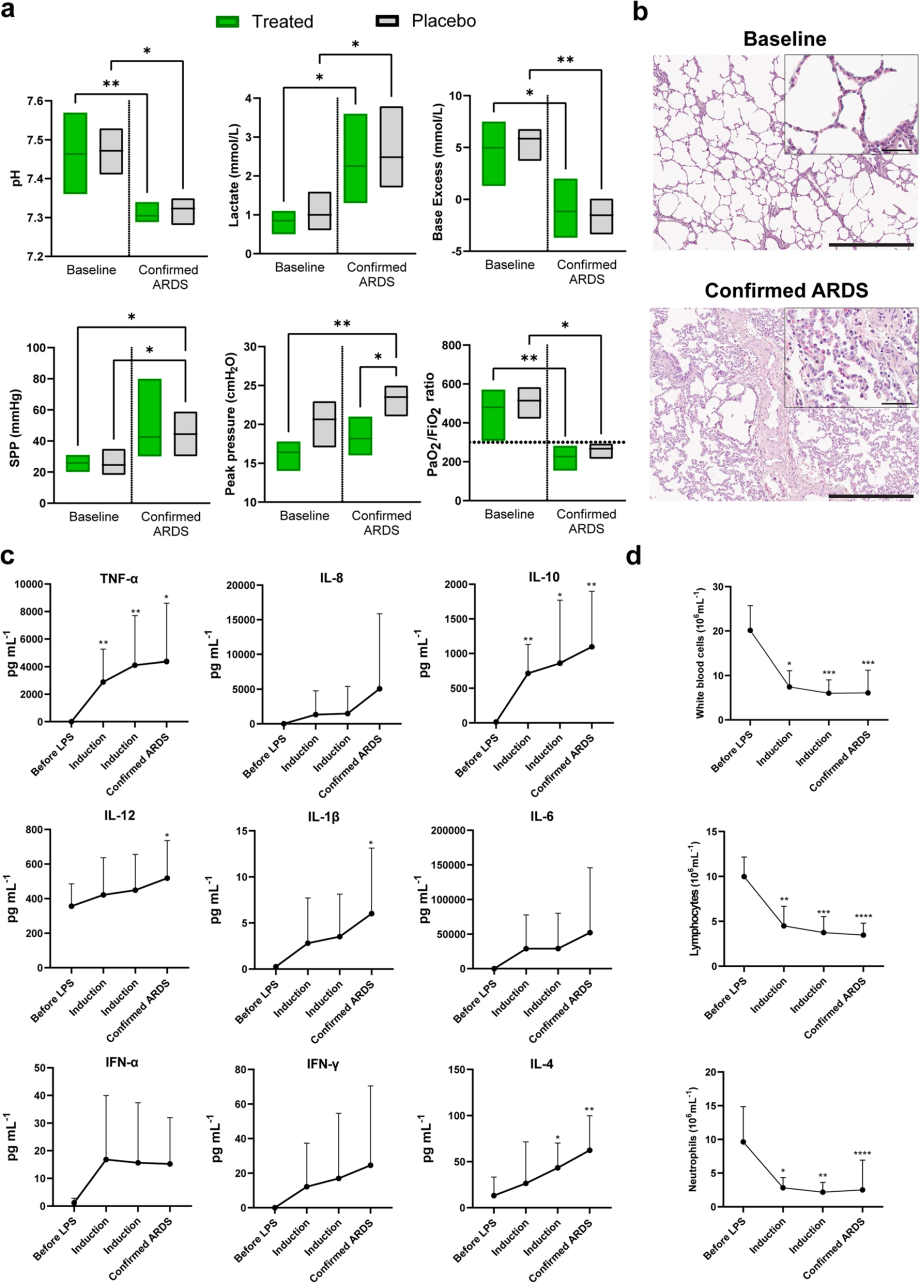
Establishment of Acute Respiratory Distress Syndrome (ARDS) lung injury. **a** Measures of pulmonary gas exchange and lung mechanics including, pH, lactate, base excess, systolic pulmonary pressure (SPP), peak pressure and PaO_2_/FiO_2_ ratio. **b** Representative haematoxylin and eosin (H&E) staining of a healthy and an ARDS porcine lung. Scale bars in the larger images represent 0.5 mm and 0.05 mm in the callouts that show a magnified portion of the tissue. **c** Cytokine measurement in plasma before LPS was administered, during two consecutive timepoints in the induction phase and at the state of confirmed ARDS lung injury (n = 12). **d** White blood cell, lymphocyte, and neutrophil counts were recorded at baseline, during lung injury induction after LPS administration (two consecutive timepoints) and at the time of confirmed ARDS (n = 12). Statistically significant differences between groups were tested with the two-sided Mann–Whitney test or the Kruskal–Wallis test when data were not normally distributed. A mixed effects-analysis with Dunnett’s multiple comparisons test was used in case of missing values. *p < 0.05, **p < 0.01, ***p < 0.001, ****p < 0.0001. Data are presented as mean ± standard deviation unless otherwise stated

Arterial blood gas, hemodynamic parameters and ventilatory parameters were measured continuously throughout the experiment as detailed in the experimental procedures and shown in Table 1, demonstrating important functional changes as ARDS developed following the endotracheal and intravenous administration of LPS. All pigs developed an ARDS and both pH and arterial oxygen saturation (SaO_2_) decreased between the baseline measurements and those taken at the time of ARDS confirmation. In the treated group, the pH fell from 7.46 ± 0.07 to 7.30 ± 0.02 (p = 0.008) while the placebo group fell from 7.47 ± 0.05 to 7.32 ± 0.03 (p = 0.045, Fig. 1a). In SaO_2_, the treated group decreased from a mean of 99.8% ± 0.4 to 97.3% ± 2.9 (p = 0.044) while the placebo group decreased from 100.0% ± 0.0 to 98.8% ± 1.0 (p = 0.094). The lactate levels significantly increased by the time of confirmed ARDS (treated group p = 0.019, placebo group p = 0.032, Fig. 2a). Base excess significantly fell in the treated group from 4.98 ± 2.48 mmol/L to − 1.17 ± 2.36 mmol/L (p = 0.048) and in the placebo group from 5.85 ± 1.12 mmol/L to − 1.53 ± 1.16 mmol/L (p = 0.010, Fig. 2a). PaCO_2_ also increased by the time of confirmed ARDS although this was not a statistically significant difference (treated group p = 0.315, placebo group p = 0.817).

**Table 1:** Clinical measurements of vitals during establishment of LPS-induced ARDS for treated subjects (gray, n = 6) and placebo (n = 6): oxygen saturation (SpO_2_, %), heart rate (HR, beats per minute, bpm), mean arterial pressure (MAP, mmHg), central venous pressure (CVP, mmHg); hemodynamic variables: mean pulmonary pressure (MPP, mmHg), pulmonary artery wedge pressure (Wedge, mmHg), cardiac index (CI, L/min), systemic vascular resistance index (SVRI, dynes s/cm^5^ m^2^), pulmonary vascular resistance index (PVRI, dynes s/cm^5^ m^2^); blood gas parameters: pH, partial pressure of carbon dioxide (pCO_2_, mmHg), lactate (mmol/L), base excess (BE, mmol/L), mechanical ventilator settings with volume-controlled ventilation: minute volume (MV, L), peak inspiratory pressure (cmH_2_O), dynamic compliance (mL/cmH_2_O), ratio of partial pressure of oxygen to fraction of inspired oxygen (PaO_2_/FiO_2_, mmHg)

	Baseline	60 min	120 min	Confirmed ARDS
Saturation, SpO_2_ (%)	97.8 ± 1.6	96.3 ± 2.9	97.3 ± 0.6	96.5 ± 0.8
	99.2 ± 0.8	99.2 ± 1.2	93 ± 11.8	95.8 ± 5.5
HR (bpm)	80.3 ± 17.0	115.5 ± 21.0	112.0 ± 28.5	121.2 ± 16.8
	105.0 ± 23.8	93.3 ± 14.7	112.8 ± 20.1	112.3 ± 16.6
MAP (mmHg)	101.5 ± 18.6	112. ± 23.1	85 ± 24.1	96 ± 34.7
	105.2 ± 10.5	102 ± 12.6	75 ± 18.1	83.8 ± 33.4
CVP (mmHg)	5.7 ± 3.1	5.5 ± 3.3	4 ± 2.7	4.8 ± 3.1
	3.8 ± 1.6	6.5 ± 2.8	4.2 ± 1.92	5.5 ± 4.1
MPP (mmHg)	19.8 ± 2.9	35.2 ± 10.9	35.3 ± 15.4	30.2 ± 13.4
	19.5 ± 6.8	25.2 ± 7.7	24.6 ± 5.6	32.7 ± 8.1
Wedge (mmHg)	11 ± 2	10.7 ± 2.7	9 ± 2.7	9.5 ± 2.1
	9.5 ± 2.4	9.17 ± 2.99	8 ± 2	10 ± 3.41
CI (L/min/m^2^)	3.5 ± 1.0	3.9 ± 1.2	4.6 ± 1.0	4.3 ± 1.1
	4.1 ± 1.7	4.4 ± 1	5.1 ± 1.9	4.6 ± 1.9
SVRI (dynes s/cm^5^ m^2^)	2384.8 ± 929.1	2309.7 ± 1103	2068.3 ± 1165.5	1752.7 ± 1211.6
	2362.8 ± 1255.3	1834.8 ± 479.7	1269.4 ± 468.7	1551 ± 543.5
PVRI (dynes s/cm^5^ m^2^)	235.5 ± 85.4	620.3 ± 492.1	447 ± 194.0	497.2 ± 540.6
	262.2 ± 90.2	306.2 ± 69.2	289 ± 99.4	462.7 ± 209.0
pH	7.46 ± 0.07	7.39 ± 0.07	7.36 ± 0.05	7.30 ± 0.02
	7.47 ± 0.05	7.42 ± 0.04	7.36 ± 0.03	7.32 ± 0.03
PaCO_2_ (mmHg)	44.2 ± 8.7	49.4 ± 8.2	49.3 ± 5.3	51.6 ± 5.5
	40.6 ± 5.9	43.7 ± 2.8	45.8 ± 1.9	47.5 ± 1.5
Lactate (mmol/L)	0.85 ± 0.21	1.18 ± 0.24	2.23 ± 0.6	2.25 ± 0.87
	1 ± 0.38	1.2 ± 0.35	1.83 ± 0.32	2.48 ± 0.79
BE (mmol/L)	4.98 ± 2.48	3 ± 2.89	0.68 ± 3	-1.17 ± 2.36
	5.85 ± 1.12	3.6 ± 2.05	0.63 ± 1.55	-1.53 ± 1.16
MV (L)	5.57 ± 0.61	5.68 ± 0.79	6.17 ± 1.61	5.93 ± 1.34
	5.48 ± 0.43	5.57 ± 0.58	6.12 ± 0.55	6.33 ± 0.58
Peak inspiratory pressure (cmH_2_O)	16.4 ± 1.4	19.2 ± 2.45	20 ± 1.73	20.63 ± 2.08
	18.17 ± 1.94	20.33 ± 2.42	21.4 ± 2.7	23.5 ± 1.76
Dynamic compliance (mL/cmH_2_O)	22.09 ± 8.07	17.49 ± 4.81	21.67 ± 4.16	16.51 ± 4.6
	21.83 ± 4.07	19.17 ± 3.25	18.2 ± 2.86	15.67 ± 2.07
PaO_2_/FiO_2_ (mmHg)	480.25 ± 94.33	374 ± 82.54	281.97 ± 92.69	227.08 ± 52.82
	514.36 ± 56.50	469.75 ± 46.78	340.5 ± 59.89	267.25 ± 29.17

Table 1 reports the hemodynamic, vitals, and pulse oximetry changes observed though many were not statistically significant. Pulmonary pressure was higher following LPS administration, with significantly higher SPP found in the placebo individuals between baseline and confirmed ARDS (SPP baseline of 24.50 ± 6.60 to confirmed ARDS of 44.33 ± 9.87, p = 0.0190, Fig. 2a).

In mechanical ventilation, higher peak pressures were observed (treated group p = 0.128, placebo group p = 0.0312, Fig. 2a) and lung compliance fell, though this did not reach statistical significance (treated group p > 0.99, placebo group p = 0.283).

The PaO_2_/FiO_2_ ratio decreased from baseline for both groups (Fig. 2a), with the treated group decreasing from 480.25 ± 94.33 mmHg to a confirmed ARDS value of 227.08 ± 52.82 mmHg (p = 0.0076) and the placebo group decreasing from 514.36 ± 56.50 mmHg to 267.25 ± 29.17 mmHg (p = 0.0478).

The ARDS state was also retrospectively evaluated by comparison of H&E stained biopsies, with representative images of healthy lungs compared to ARDS-damaged lungs shown in Fig. 2b.

### Cytokine levels increased and blood cell counts decreased following LPS administration

Several cytokines were increased over the course of ARDS induction (Fig. 2c), including TNF-α which rose from 1.34 ± 1.13 pg/mL to 4372.85 ± 4230.56 pg/mL (p = 0.0114) and IL-12 which rose from 356.40 ± 129.25 pg/mL to 518.40 ± 217.23 pg/mL (p = 0.0437). Other cytokines which significantly increased included IL-β (from 0.25 ± 0.13 pg/mL to 6.02 ± 7.10 pg/mL p = 0.0426) and IL-4 (from 13.19 ± 20.11 pg/mL to 62.30 ± 37.55 pg/mL, p = 0.0046). IL-10 increased significantly from 13.04 ± 20.41 pg/mL to 1095.70 ± 803.31 pg/mL (p = 0.0018). Several other cytokines increased by ARDS confirmation but were not found to be statistically significant, including IL-8, IL-6, IFN-α and IFN-γ.

After the induction of ARDs with LPS, white blood cell counts in total were found to significantly decrease by the time ARDS was confirmed (Fig. 2d). Baseline samples had an average of 20.14 ± 5.58 × 10^6^ cells/mL and fell to 6.08 ± 5.13 × 10^6^ cells/mL by confirmed ARDS (p = 0.0001). Within those samples, lymphocyte counts decreased from a baseline of 9.98 ± 2.18 × 10^6^ cells/mL to a confirmed ARDs value of 3.47 ± 1.32 × 10^6^ cells/mL (p < 0.0001) and neutrophil counts decreased from 9.62 ± 5.24 × 10^6^ cells/mL to 2.50 ± 4.43 × 10^6^ cells/mL (p < 0.0001).

### Integrin α10β1-MSC-treated group required less inotropic support

**Fig. 3:**
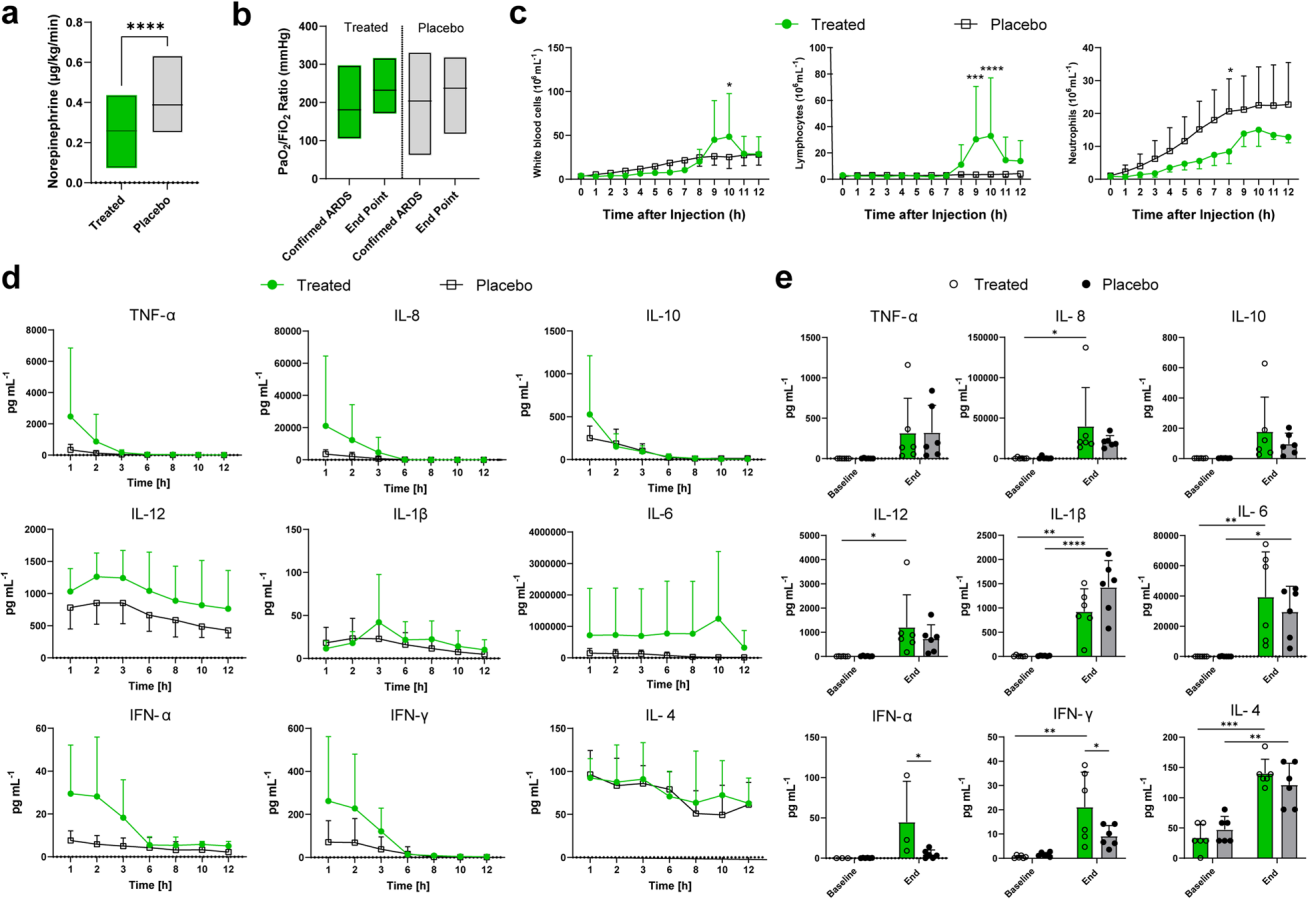
Improved ARDS state following MSC administration vs placebo. **a** Amounts of norepinephrine (µg/kg/min) administered as inotropic support to support hemodynamic stability. **b** PaO_2_/FiO_2_ ratio was quantified at the time of confirmed ARDS and at the end-point for both groups. **c** Cell counts for white blood cells, lymphocytes and neutrophils were analysed. Statistical significance applies to direct comparison of the treated to the placebo group (mixed effects model Sidak’s multiple comparisons test). **d** Plasma cytokine levels monitored after the administration of treatment or placebo. **e** Bronchoalveolar lavage fluid (BALF) cytokine levels were tested at baseline and at the endpoint of the experiment. Statistically significant differences between groups were tested with two-sided Student’s T-test and within groups with ANOVA when data were normally distributed. The two-sided Mann–Whitney test and the Kruskal–Wallis test were used when data were not normally distributed. Multiple two-sided Mann–Whitney tests were used to compare the groups when data were not normally distributed. Mixed-effects analysis with Šídák’s multiple comparisons test was used to compare timepoints within each group, *p < 0.05, **p < 0.01, ***p < 0.001, ****p < 0.0001. Data are presented as mean ± standard deviation unless otherwise stated. Analyses were conducted on treated group (n = 6) and placebo group (n = 6)

ARDS confirmation was followed by either the integrin α10β1-MSC treatment or a placebo infusion. Table 2 shows vitals, arterial blood gas, hemodynamic parameters and ventilatory parameters for both groups following treatment. All individuals required inotropic support with norepinephrine throughout the experimental timeline following the induction of ARDS. The integrin α10β1-MSC treated group required significantly less norepinephrine 0.44 ± 0.49 µg/kg/min by the end of the experimental timeline, compared to the placebo group requiring 0.65 ± 0.57 µg/kg/min (p < 0.0001) (Fig. 3a). Within measures from the blood gases, there were initial differences between the pH, base excess, and lactate within the first 120 min though these were not statistically significant differences (Additional file 1: Fig. S2). By the end of the experiment, however, the treated and placebo groups shared similar values. As a measure of gas exchange, the PaO_2_/FiO_2_ ratio was continuously monitored and was improved for both groups. There was no statistical difference between groups by the end of the experiment (Fig. 3b). The treated group improved from an average of 180.4 ± 77.8 mmHg at the time of administration to 203.2 ± 120.1 mmHg (p = 0.9004) while the non-treated group 232.3 ± 54.4 mmHg to 273.5 ± 55.5 mmHg (p = 0.3680) by the end of the experiment.

**Table 2:** Clinical measurements of vitals during the follow-up after treatment (starting 0 min after) with MSCs (gray, n = 6) or with placebo (n = 6): oxygen saturation (SpO_2_, %), heart rate (HR, beats per minute, bpm), mean arterial pressure (MAP, mmHg), central venous pressure (CVP, mmHg); hemodynamic variables: mean pulmonary pressure (MPP, mmHg), pulmonary artery wedge pressure (Wedge, mmHg), cardiac index (CI, L/min/m^2^), systemic vascular resistance index (SVRI, dynes s/cm^5^ m^2^), pulmonary vascular resistance index (PVRI, dynes s/cm^5^ m^2^); blood gas parameters: pH, partial pressure of carbon dioxide (pCO_2_, mmHg), lactate (mmol/L), base excess (BE, mmol/L), mechanical ventilator settings with volume-controlled ventilation: minute volume (MV, L/min), peak inspiratory pressure (cmH_2_O), dynamic compliance (mL/cmH_2_O), ratio of partial pressure of oxygen to fraction of inspired oxygen (PaO_2_/FiO_2_, mmHg)

	0 min	60 min	120 min	240 min	360 min	480 min	600 min	End Point (EP)
Saturation, SpO_2_ (%)	93.8 ± 1.2	94.3 ± 2.6	95 ± 2.8	95 ± 1.7	94 ± 1.9	95.7 ± 4.0	97 ± 1	95.2 ± 2.5
	95.8 ± 3.1	94.5 ± 4.0	96.3 ± 2.7	95.4 ± 2.4	95.4 ± 4.2	96 ± 3.5	96.2 ± 5.8	96.7 ± 4.4
HR (bpm)	121.3 ± 8.9	124.7 ± 8.6	124.8 ± 13.2	117.6 ± 15.5	111 ± 11.4	108.7 ± 9.0	99 ± 7.9	105.2 ± 14.3
	133.5 ± 42.6	145.3 ± 51.4	137.3 ± 48.2	132.2 ± 40.5	116 ± 20.0	110 ± 19.9	112 ± 19.7	109.7 ± 22.9
MAP (mmHg)	80.7 ± 20.1	77.7 ± 18.7	70.8 ± 16.6	74.4 ± 16.1	70.4 ± 16.4	68.7 ± 12.9	66.0 ± 18.2	57.2 ± 10.3
	78.8 ± 29.2	80.3 ± 29.7	77.5 ± 26.8	92.0 ± 11.7	75.0 ± 8.2	75.4 ± 9.7	65.6 ± 8.4	57.8 ± 10.4
CVP (mmHg)	5.8 ± 3.0	4.8 ± 2.7	5.5 ± 2.5	7 ± 3.2	9.4 ± 4.7	7 ± 2.7	6.7 ± 4.7	10.2 ± 3.9
	5.8 ± 4.1	6.7 ± 3.9	6.5 ± 2.8	8.4 ± 3.2	7.4 ± 2.6	8.6 ± 3.9	9 ± 4.2	9.8 ± 4.2
MPP (mmHg)	33.8 ± 10.9	28.3 ± 8.6	25.8 ± 8.2	27.8 ± 9.2	34 ± 7.5	25 ± 6.1	24.3 ± 2.1	28.7 ± 9.0
	33.7 ± 7.5	34.3 ± 6.4	29.8 ± 6.8	27.2 ± 5.2	24.6 ± 8.8	22.4 ± 6.9	23 ± 6.4	23.7 ± 6.8
Wedge (mmHg)	10.0 ± 3.4	8.2 ± 1.8	8.3 ± 2.8	9.6 ± 4.3	15.8 ± 7.2	9.7 ± 2.3	10.3 ± 1.5	13.5 ± 6.9
	9.0 ± 3.3	9.2 ± 2.6	10.7 ± 2.9	11.6 ± 5.0	12.6 ± 10.0	11 ± 3.8	11.4 ± 4.3	11.5 ± 4.5
CI (L/min m^2^)	4.7 ± 1.0	4.6 ± 1.6	4.4 ± 1.5	4.7 ± 1.2	4.5 ± 0.9	4.6 ± 0.3	4.5 ± 0.2	4.0 ± 0.7
	4.5 ± 1.0	5.0 ± 1	5.6 ± 1.9	4.7 ± 0.7	4.5 ± 1.0	4.5 ± 1.5	5.0 ± 1.8	5.3 ± 2.1
SVRI (dynes s/cm^5^ m^2^)	1594.5 ± 875.3	1425.7 ± 769.2	1440.6 ± 685.8	1216.4 ± 434.4	1020.2 ± 333.5	1144.7 ± 214.3	1109.3 ± 297.0	886.2 ± 305.1
	1244.2 ± 290.6	1138.8 ± 412.5	1231.5 ± 636.2	1470.2 ± 195.0	1249.4 ± 289.8	1275.6 ± 358.4	971.6 ± 309.1	865 ± 432.7
PVRI (dynes s/cm^5^ m^2^)	446.5 ± 333.98	413.5 ± 263.3	440.6 ± 224.8	345.2 ± 161.9	309.4 ± 85.5	292.3 ± 109.9	272 ± 60.3	299.3 ± 106.6
	433.2 ± 195.6	398.3 ± 148.4	354.7 ± 197.9	295.8 ± 145.7	241 ± 152.3	269 ± 151.6	255.4 ± 156.0	245 ± 138.7
pH	7.30 ± 0.05	7.34 ± 0.08	7.34 ± 0.07	7.34 ± 0.06	7.34 ± 0.04	7.35 ± 0.07	7.36 ± 0.09	7.30 ± 0.09
	7.29 ± 0.03	7.27 ± 0.07	7.30 ± 0.04	7.33 ± 0.03	7.31 ± 0.08	7.34 ± 0.03	7.34 ± 0.03	7.31 ± 0.09
PaCO_2_ (mmHg)	53.7 ± 5.7	49.7 ± 5.3	47.5 ± 5.7	46.2 ± 6.3	46.7 ± 5.5	42.9 ± 2.9	40.4 ± 2.6	46.1 ± 6.3
	49.1 ± 2.1	51.4 ± 9.2	47.3 ± 3.1	44.7 ± 4.9	44.4 ± 6.3	42.5 ± 4.1	42.7 ± 3.7	43.1 ± 4.5
Lactate (mmol/L)	2.37 ± 0.53	2.93 ± 0.96	3.4 ± 0.99	3.4 ± 2.05	3.12 ± 1.89	2.17 ± 1.39	2.33 ± 1.64	3.22 ± 1.78
	2.9 ± 0.81	4.27 ± 1.91	5.03 ± 3.57	2.84 ± 2.32	1.9 ± 1.5	2.9 ± 1.57	2.96 ± 1.48	4.53 ± 4.57
Base excess (mmol/L)	− 0.87 ± 4.25	0.08 ± 4.02	− 1.38 ± 3.93	− 1.66 ± 4.59	− 1.76 ± 3.73	− 1.77 ± 4.03	− 2 ± 4.5	− 3.68 ± 3.25
	− 2.87 ± 1.45	− 3.38 ± 2.09	− 3.37 ± 2.51	− 3.02 ± 0.33	− 3.82 ± 2.84	− 2.64 ± 2.18	− 2.66 ± 2.24	− 4.25 ± 4.06
MV (L/min)	6.07 ± 1.32	6.87 ± 1.37	7.63 ± 1.31	7.78 ± 0.75	7.88 ± 0.87	8.37 ± 1.72	8.7 ± 1.2	7.92 ± 1.32
	6.58 ± 0.34	6.48 ± 3.01	7.73 ± 1.29	7.62 ± 0.92	7.94 ± 0.86	8.12 ± 0.52	8.34 ± 0.77	8.47 ± 0.78
Peak inspiratory pressure (cmH_2_O)	24.3 ± 3.18	25.23 ± 1.91	25.42 ± 1.69	24.02 ± 1.41	24.8 ± 2.59	23.33 ± 1.15	25.33 ± 3.21	27.73 ± 1.88
	24.17 ± 2.79	25 ± 3.29	24 ± 4.05	23.4 ± 2.3	22.6 ± 1.14	23.4 ± 2.07	23.2 ± 2.28	24.17 ± 3.76
Dynamic compliance (mL/cmH_2_O)	14.72 ± 5.89	13.8 ± 5.11	13.73 ± 4.44	15.71 ± 3.02	15.4 ± 3.36	16.67 ± 2.08	15.33 ± 3.21	12.76 ± 4.13
	15 ± 2.53	15 ± 2.97	15.83 ± 3.06	15.8 ± 2.39	16.4 ± 1.52	16 ± 1.87	16.2 ± 1.92	15.33 ± 2.73
PaO_2_/FiO_2_ (mmHg)	180.86 ± 77.49	156.36 ± 60.22	171.72 ± 80.26	172.97 ± 63.2	152.95 ± 95.6	195.63 ± 88.19	243.15 ± 75.81	203.83 ± 120.39
	232.25 ± 54.39	208.67 ± 70.92	209.17 ± 66.08	214.15 ± 59.88	219.15 ± 70.57	242.62 ± 90.55	250.51 ± 66.1	237.34 ± 79.87

### Cell counts and cytokine levels decreased after integrin α10β1-MSC administration

Total circulating white blood cell counts were similar across the experimental follow-up between the treated and placebo groups, with elevated counts noted in the treated individuals by the 10th hour of the post-treatment follow-up (treated group 48.50 ± 49.22 × 10^6^ cells/mL, placebo 25.32 ± 13.01 × 10^6^ cells/mL, p = 0.0425) (Fig. 3c). There were more circulating lymphocytes in the treated group in the latter half of follow-up while neutrophil counts were decreased. Neutrophil counts in the two groups diverged after 2 h of follow-up, with the placebo group increasing in count at a greater rate than the treated. At 8 h of follow-up, the number of neutrophils in the treated group (8.40 ± 3.66 × 10^6^ cells/mL) was found to be statistically lower than the placebo group (20.66 ± 9.90 × 10^6^ cells/mL, p = 0.0493).

In terms of cytokines detected in the plasma, all groups demonstrated a similar decline from a peak at the time of ARDS confirmation and cell/placebo administration to the end of the experiment (Fig. 3d). A relatively higher rate of decline from higher starting levels is noted for IFN-α and IFN-γ in the treated groups. Within the BAL, certain cytokines were elevated by the end the experiment compared to the start, including IL-8, IL-12, IL-1β, IL-6, IFN-γ, and IL-4. IL-1β was lower in the BAL of the treated group compared to the placebo group (Fig. 3e). These samples were compared to their own baseline values (Fig. 3e).

### Reduced hypercoagulopathy in the treated group receiving integrin α10β1-MSC

ROTEM data showed analyses for extrinsically activated assays with tissue factor (EXTEM), intrinsically activated assays using phospholipid and ellagic acid (INTEM) as well as intrinsically activated assays with the addition of heparinase (HEPTEM). All samples were collected in a similar fashion using identical sodium citrate vacutainer tubes, however, there were two samples which were unable to be analysed from the same placebo individual: one at 2 h post-placebo and the other at 6 h post-placebo treatment. Despite adequate coagulation, the sample clotted immediately, and the machine was unable to read it. The samples from those time points were resultantly removed from data analysis.

**Fig. 4:**
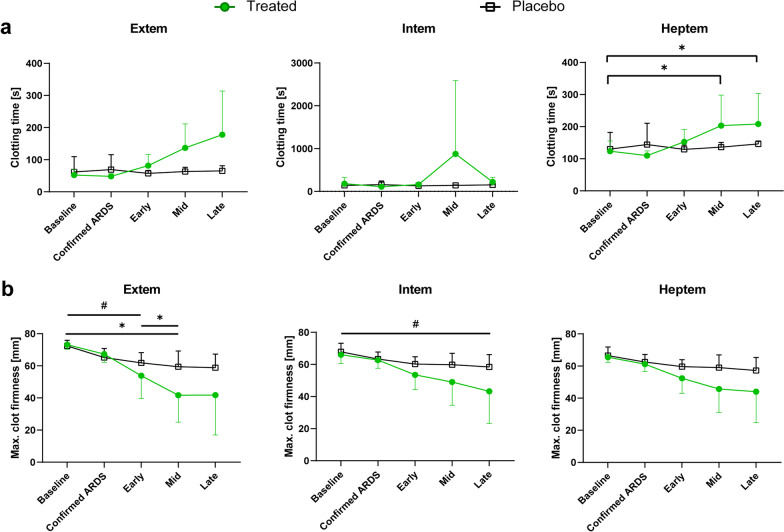
Monitoring of haemostasis by rotational thromboelastometry (ROTEM). Comparison of extrinsic (EXTEM) and intrinsic (INTEM) clotting parameters, and heparin modifications (HEPTEM) throughout the experiment. Samples were analysed before administration of lipopolysaccharide, after confirmed ARDS and in the early, mid, and lates phases of the monitoring following MSC or placebo administration, respectively. **a** Clotting time in seconds and **b** Maximum clot firmness in mm are shown for the treated group (green circles) and the placebo group (black squares). Multiple two-sided Mann–Whitney tests were used to compare the groups when data were not normally distributed. Mixed-effects analysis with Šídák’s multiple comparisons test was used to compare timepoints within each group. *p < 0.05 for comparisons within the treated group, ^#^p < 0.05 for comparisons within the placebo group. Data are presented as mean ± standard deviation unless otherwise stated. Analyses conducted on treated group (n = 6) and placebo group (n = 6)

Within the remaining cohort, there were no significant differences in the clotting times (CT), maximum clot firmness (MCF) and the clot formation times (CFT) in any of the assays between the baseline samples and the early phase samples taken after cell or placebo administration (Fig. 4a, b, Additional file 1: Fig. S3a). In the follow-up, the treated group had increasingly longer clotting times by the end of the monitoring period post-treatment, including within the EXTEM, INTEM, and HEPTEM assays (Fig. 4a). This was a significant increase by the mid-phase (p = 0.0128) and late-phase (p = 0.0167) monitoring in HEPTEM. The placebo group maintained a consistent clotting time to the end of the experiment. MCF values were numerically higher in the placebo group compared to the treated group, however, these differences did not reach statistical significance (Fig. 4b). A10 values representing clot firmness in millimetres after 10 min also were higher in the placebo group in the EXTEM and HEPTEM assays, although this was not statistically significant (Additional file 1: Fig. S3b).

### Fewer signs of severe ARDS in histological characterization in the treated group

**Fig. 5:**
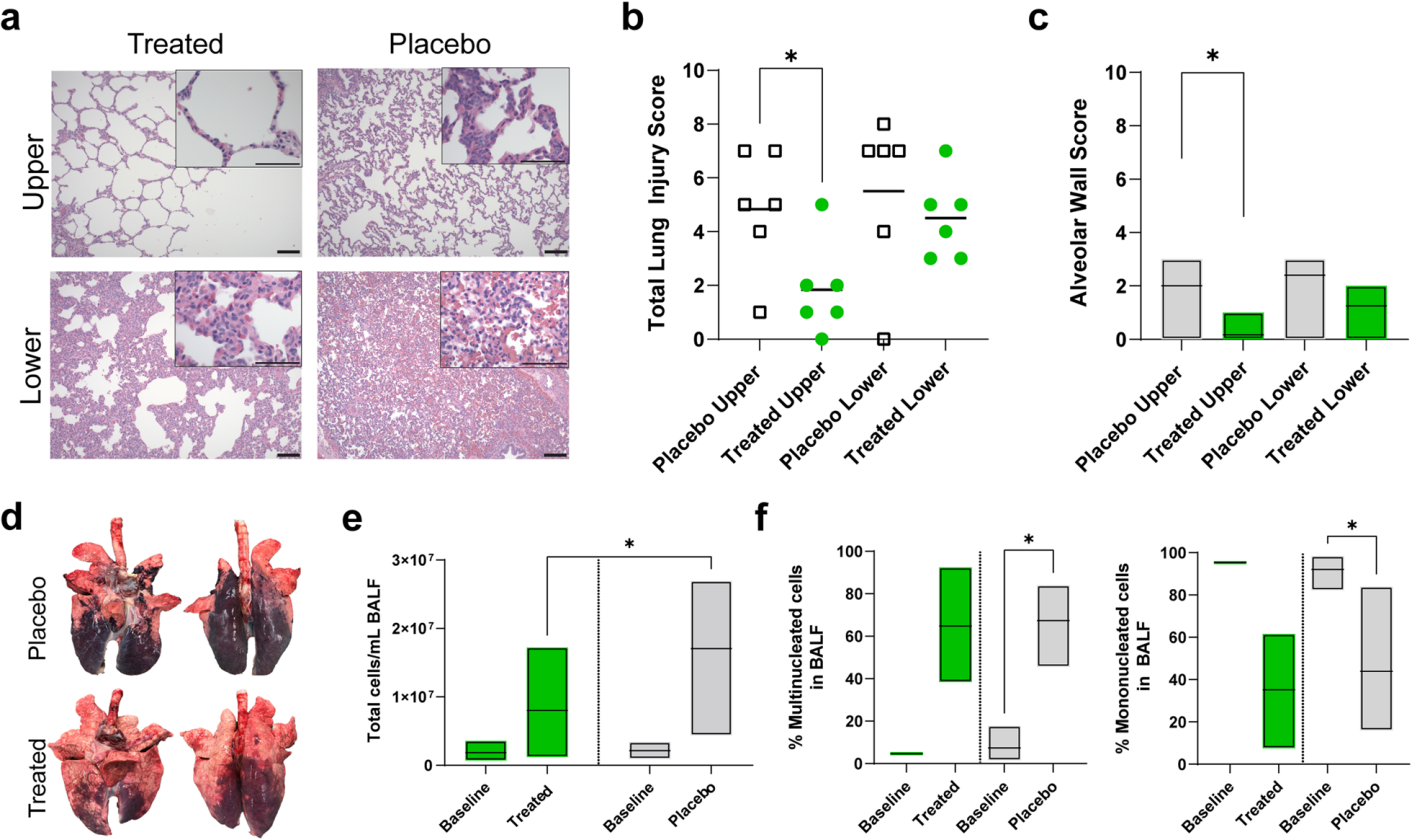
Histopathological and bronchoalveolar lavage fluid analysis. **a** Representative haematoxylin and eosin (H&E) staining of biopsies from the upper and lower lobes are shown for the treated and placebo groups. Scale bars in the larger images represent 0.1 mm and 0.05 mm in the callouts that show a magnified portion of the tissue. **b** Lung injury scoring by a blinded pathologist was generated for biopsies taken at the endpoints. **c** Alveolar wall thickening scores by a blinded pathologist are shown for biopsies taken at the endpoints. **d** Images of gross morphology for a treated and placebo lung (front and back view) at the endpoint of the experiment. **e** Bronchoalveolar lavage fluid (BALF) was collected and the total cells/mL were measured for all individuals. **f** BALF immune cell counts were quantified across 200–300 cells per sample and grouped into relative percentages by mononuclear and multinuclear cells. Data are presented as mean with min and max. Statistically significant differences between groups were tested with two-sided Student’s *T*-test and within groups with ANOVA when data were normally distributed. The two-sided Mann–Whitney test and the Kruskal–Wallis test were used when data were not normally distributed, *p < 0.05, **p < 0.01, ***p < 0.001. All values represent the mean ± standard deviation unless otherwise stated

Histological evaluation with H&E staining of lung biopsies taken from the upper and lower lobes at the end of the experiment demonstrated signs of lung injury (Fig. 5a). Other signs of ARDS-induced damage included infiltration of immune cells, including high levels of alveolar macrophages, haemorrhage, acute and chronic inflammation within individuals in the placebo group. Blinded scoring was performed on all pigs at the end of the experiment on the upper and lower lobes, showing significantly less lung tissue injury among the integrin α10β1-MSC treated upper lobes (Fig. 5b). Scores included an average of 1.83 ± 1.72 in the treated group’s upper lobes, which was significantly lower than the score of 4.83 ± 2.23 in the placebo group’s upper lobes (p = 0.0261, Fig. 5b). Scoring of alveolar wall thickening in the upper lobes showed significantly greater distribution in the placebo group (2.00 ± 1.23) compared to the treated group (0.17 ± 0.41, p = 0.007, Fig. 5c). Gross morphology shows the degree of injury due to the ARDS as seen in the explanted lungs from the end of the experiment (Fig. 5d).

### Immunophenotypically surface marker profiles in peripheral blood mononuclear cells remained similar between the groups

PBMCs were analysed for changes in surface marker profiles, the data for which are shown in Additional file 1: Fig. S1. There were no significant changes identified between the treated and placebo groups over the experimental timeframe.

### Wet/dry weight ratio showed similar levels of edema

Between the treatment and the placebo groups, the ratio of wet to dry weight in lung biopsies was found to not be statistically different between each other (Additional file 1: Fig. S4). The treatment group had an average ratio of 3.48 ± 0.80 while the placebo group had an average ratio of 2.90 ± 0.37 (p = 0.3939).

### BALF analysis showed higher cell counts at the end of the experiment

Significantly higher numbers of total cells per mL were observed in the BALF at the end of the experiment in the placebo group compared to the treated group. The placebo group were found to have 1.71 × 10^7^ ± 9.37 × 10^6^ cells/mL relative to the 8.02 × 10^6^ ± 6.76 × 10^6^ cells/mL in the treated group (p = 0.0420, Fig. 5e). Relative percentages of multinucleated and mononuclear cells were quantified within stained slides of BALF (Fig. 5f, Additional file 1: Fig. S5). By the end of the experiment, integrin α10β1-MSC-treated samples had higher percentages of multinucleated cells (62.28% ± 24.87%) compared to baseline samples (6.60% ± 2.14%, p = 0.0625) which was similar to the finding in the placebo samples (65.80% ± 18.31%) compared to their own baseline (9.65% ± 6.84%, p = 0.0312). Consequently, the percentage of mononuclear cells decreased and by the experimental end, both the integrin α10β1-MSC-treated group (37.72% ± 24.87%) and the placebo group mononuclear cells (34.10% ± 18.31%) were lower than their respective baselines (integrin α10β1-MSC-treated baseline 93.34% ± 2.14% p = 0.0625, placebo baseline 90.35% ± 6.84%, p = 0.0312).

### There were no differences in survival between groups

**Fig. 6:**
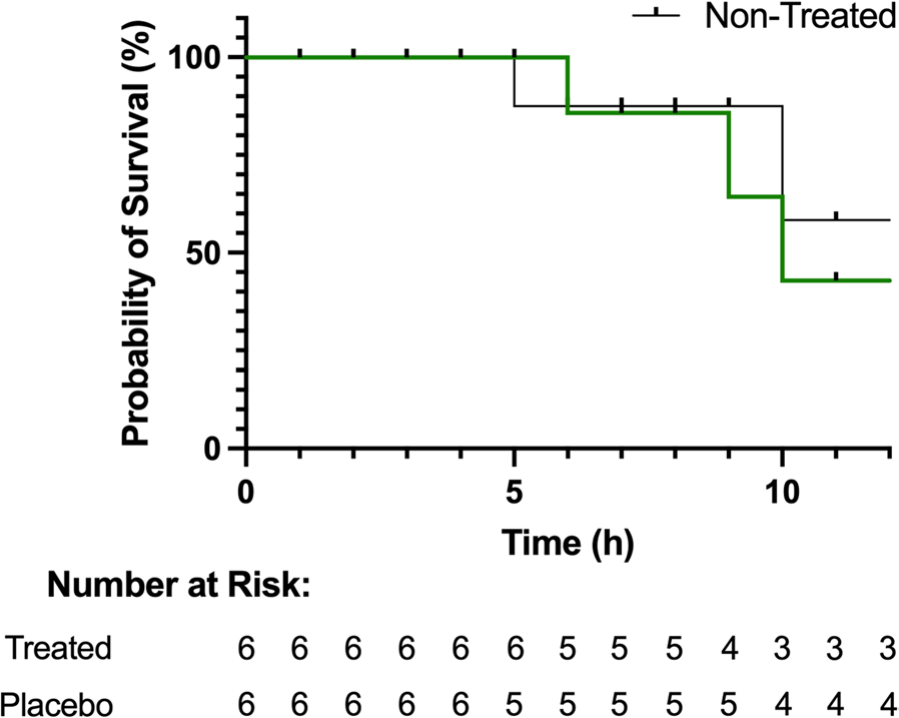
Survival in the treated and placebo groups. The Kaplan–Meier curve shows the probability of survival in the treated group (green) and the placebo group (black) over 12 h with the number at risk for each timepoint (log-rank p = 0.6410)

Following monitoring after the induction of ARDS and the administration of treatment, all subjects survived to at least 5 h (Fig. 6). One individual in the placebo group survived until 5 h and another to 10 h post-placebo. In the treatment group, one survived until 6 h, another to 9 h, and a third to 10 h. There were no significant differences in the mortality rates between the two groups (log-rank p = 0.6410).

### Precision lung cut slices (PCLS) allowed for continuation of experimental treatment groups

At the end of the experimental timeline, the ARDS-damaged lungs from both treated and placebo groups were explanted and processed to generate PCLS (Additional file 1: Fig. S6a). This facilitated continued monitoring of the treatment effect on the tissue in an ex vivo method without having to maintain the severe in vivo injury within the model organism. These punches kept in culture over the course of 5 days showed increasing histological improvements in both groups compared to baseline histology samples taken immediately at the experiment’s end (Additional file 1: Fig. S6b, c). Modified lung injury scores were highest in the baseline samples for both the treated and placebo groups and both improved by 24 h in culture. This improvement was maintained throughout the culture period. Injury scores were found to be significantly lower in the integrin α10β1-MSC treated PCLS on day 5 compared to the placebo baseline (treated day 5 score of 6.83 ± 1.00 vs placebo baseline 21.58 ± 4.19, p = 0.0396).

## Discussion

In this study, integrin α10β1-MSCs derived from human adipose tissue were administered to pigs with LPS-induced ARDS to determine the treatment effects and safety of integrin α10β1-MSCs on lung injury. The results show that MSC treatment was associated with decreased need for inotropic support over the experimental timeline along with an anticoagulative effect compared to the placebo group. Histological examination of biopsies taken from the treated group also showed fewer signs of injury. There were, however, no significant differences in pulmonary gas exchange based on the PaO_2_/FiO_2_ ratio between groups. As demonstrated in previous studies, the administration of MSCs in this large animal model is safe without any adverse events over the monitoring period.

ARDS here was induced via a dual-hit of endotracheal and intravenous LPS. LPS causes endothelial damage within the lung, stimulating systemic inflammatory responses and programmed cell death in the endothelium [19]. The use of LPS recapitulates the pathophysiology of clinical ARDS and generates a sepsis-like state which can be used to test potential treatment modalities [18, 20, 21]. LPS is used for ARDS animal models due to its specific targeting of alveolar type II cells and pulmonary surfactant, which also leads to damage that mimics the neutrophil response seen in human [22, 23]. We have demonstrates the use of this sepsis-like injury state in previous porcine lung injury models [21, 24]. This results in a reliable and consistent model of sepsis-like disease in animals without overwhelming septicaemia. ARDS was confirmed with oxygenation ratios as well as retrospectively via histological examination of lung tissue biopsies. There were no significant differences in disease severity between the treated and placebo groups at time of confirmed ARDS.

Once ARDS had been confirmed, integrin α10β1-MSCs were given intravenously to half of the pigs in a randomised fashion. In line with previous findings, the use of MSCs in this context did not lead to adverse hemodynamic or respiratory events over the monitoring period. Observed effects of treatment included a significantly decreased need for inotropic support. Administration of norepinephrine, as was needed in this study, is an independent predictor of death in human ARDS patients [25] and norepinephrine dosages have been significantly higher in ICU non-survivors compared to survivors [26]. MSC therapy has previously been demonstrated to correlate with lower vasopressor use within large animals [27]. This study additionally found fewer circulating neutrophils in the treated group. Previous studies have also demonstrated an immunomodulatory effect following MSC treatment, including alterations in neutrophil migration [28].

Following histological staining of biopsies, lung injury scoring in the treated group revealed a significantly lower degree of damage in the upper lobes compared to the placebo group. Other preclinical models of treatment of lung injury used higher cell doses (300 × 10^6^ in a mean weight of 52.6 kg), and also found lower lung injury scores upon histological examination [27]. Improvements in histological signs of injury were found in this study with a lower dose of 5 × 10^6^ cells/kg. The biopsies of the placebo group in this study also showed greater alveolar wall thickening and capillary congestion appreciated on a microscopic level than the treated group, and areas of widespread haemorrhage could be seen on macroscopic evaluation of the lungs.

Integrin α10β1-MSC treatment in this study was also observed to have an anticoagulative influence in the ROTEM results. When examining the clotting time (CT), its activation and initiation trended towards longer times both in the INTEM and EXTEM pathways after treatment. Maximum Clot Firmness (MCF) was decreased in the MSC treated group in both INTEM and EXTEM. Adipose-derived MSCs have previously demonstrated prolonged clotting times when given at a dose of 10 × 10^6^ cells/kg in rats with a similar decline in MCF as observed in this porcine study [29]. This anticoagulative effect may have important implications given the degree of hypercoagulation in the placebo group. One individual became hypercoagulable to the extent that samples taken 2- and 6-h post-placebo could not be measured despite appropriate anticoagulation in the blood tube with sodium citrate. Regulation of haemostasis is important in the context of COVID-19-induced ARDS coagulopathies, which is attributed to inflammation and infection severity [30, 31]. Infection with COVID-19 already carries a significant risk of thrombosis in patients, even following anticoagulation [32] and abnormal coagulation has been correlated to massive microvascular clotting within the lungs [33]. The MSCs in this study demonstrated favourable effects on both the intrinsic and extrinsic coagulation cascade, which could help in the management of ARDS coagulopathies.

As in this investigation, human clinical trials have not yet demonstrated improvements in oxygenation levels despite preclinical evidence that MSCs improve in lung injury. In the phase 2a START trial, 10 × 10^6^ bone marrow-derived cells per kg were safely infused into ventilated patients with moderate to severe ARDS. Treatment did not reduce mortality and a non-statistically significant improvement in the oxygenation index was observed [11].

While the START trial pointed to unexpectedly diminished MSC viability after washing their cells, treatment timing could also have a greater effect on efficacy than currently appreciated. Preclinical models have focused on MSC treatment following the onset of injury, finding that MSCs are well tolerated directly after injury and can reduce extravascular lung water [8]. Mortality rates in a sepsis model of ARDS were higher when cells were given 12 h following ARDS induction compared to the same dosage given 1 h after induction [34]. The START trial aimed to deliver the treatment within the exudative phase of ARDS which was accomplished by intravenous dosing within 7 days of ARDS diagnosis, which would be a later in the disease course than preclinical models. In this study, treatment administration followed confirmation of ARDS. The modest effect of treatment may be attributed to the later timing of treatment and future studies could focus on cell administration closer to the time of pulmonary insult.

Many clinical trials have studied bone marrow derived MSCs, but cells originating from adipose tissue have also been shown to be effective immunomodulators in other settings. Benefits of adipose-derived cells include the availability of the tissue source. For these reasons, adipose-derived cells were chosen as a treatment in this study. Additionally, the cells underwent a selection step for expression of the collagen receptor and MSC marker integrin α10β1 to generate a homogenous MSC preparation, increasing consistency between donors and batches. Integrin α10β1 MSCs have previously demonstrated improved immunomodulation capacity in vitro as well as immunomodulatory and regenerative capacity in vivo [14]. The cells in this model were administered intravenously after consideration of other studies in which direct comparison of intrabronchial, intratracheal and intravenous routes all showed efficacy. Additionally, clinical trials have chosen intravenous administration [9, 11].

There are limitations in the study, including the limited follow-up time that was possible within the porcine ARDS model. Following ARDS induction, pigs were followed-up for 12 h which was chosen based on feasibility and ethical concerns. Given these difficulties, an ex vivo “continuation” model using precision cut lung slices (PCLS) was initiated to follow the disease state for longer. Lung slices produced from both the treated and the placebo group were kept in culture for 5 days and the resulting histological injury scores demonstrated that in both cases, the tissue benefited from the transition to culture medium. Further studies could explore the extent of MSC distribution within the punches to further characterize the treatment results and uncover if the MSCs can act in a disease-modulating manner over a longer period.

Other limitations to consider include the differences between a model of ARDS compared to clinical infection-induced ARDS. ARDS in this porcine model was induced quickly over a few hours and involved an intense “dual-hit” strategy with both endotracheal and intravenous toxin administration. The time from a human patient’s exposure to the infectious agent to the development of a mild or severe ARDS could be longer than the ARDS induced in this study and as a result, could affect the type of injury and the lung environment in a manner not reproduced in this model. Furthermore, the results of this study, raise an important question regarding the timing of dose administration. It is possible that the treatment outlined here was given at a point where damage had irreversibly occurred following toxin administration. Further studies could explore the differences in outcomes if the same cells are given earlier in ARDS development.

In conclusion, this placebo-controlled study shows that integrin α10β1-selected MSCs had a therapeutic effect on ARDS as shown by the decreased need for inotropic support, decreased histopathological lung tissue injury and the anticoagulative effect seen in this large animal model. The MSC treatment did not lead to any adverse events over the monitoring period, but did not result in changes to survival or oxygenation ratios. The findings of this work merit further investigation into the use that these cells have for treating ARDS, a severe disease for which no current treatment has been proven effective.

## Supplementary Information

**Additional file 1.** Supplementary methods and supplementary figures S1-S6.** Figure S1**. Flow cytometry on isolated peripheral blood mononuclear cells (PBMCs). **Figure S2**. Measures of pulmonary gas exchange and lung mechanics following administration of treatment or placebo. **Figure S3**. Monitoring of haemostasis by rotational thromboelastometry (ROTEM). **Figure S4**. Measure of the wet/dry ratio. **Figure S5**. Representative images of bronchoalveolar lavage fluid (BALF) staining. **Figure S6**. Precision cut lung slices (PCLS) continued experimental conditions in an ex vivo setting.

## Data Availability

The data sets generated and analysed in the current study are available from the corresponding author on reasonable request.
