# Supplementary material for: Integrin α10β1-selected mesenchymal stem cells reduced hypercoagulopathy in a porcine model of acute respiratory distress syndrome
Source: Respir Res. 2023 May 31;24:145. doi: 10.1186/s12931-023-02459-6 (PMC10230488; doi:10.1186/s12931-023-02459-6)
Supplement: Supplementary file 1 — Additional file 1. Supplementary methods and supplementary figures S1-S6. Figure S1. Flow cytometry on isolated peripheral blood mononuclear cells (PBMCs). Figure S2. Measures of pulmonary gas exchange and lung mechanics following administration of treatment or placebo. Figure S3. Monitoring of haemostasis by rotational thromboelastometry (ROTEM). Figure S4. Measure of the wet/dry ratio. Figure S5. Representative images of bronchoalveolar lavage fluid (BALF) staining. Figure S6. Precision cut lung slices (PCLS) continued experimental conditions in an ex vivo setting. [file 12931_2023_2459_MOESM1_ESM.docx]

**Additional file 1**

Additional methods

*Generation of Precision Cut Lung Slices (PCLS)*

Following the end of monitoring, four lungs from each group were explanted *en bloc* and flushed with sterile saline solution. These lungs were then kept at 4°C until a distal section of the lower lobes was cut and filled via a visible bronchiole with warm, low-gelling agarose (3% by weight, Sigma-Aldrich) kept at 42°C and mixed with sterile cultivation medium (DMEM/Ham’s F12; Gibco Life Science Technologies, Grand Island, New York, USA). The agarose-filled lung portion was then cooled at 4°C for 30 minutes to allow for agarose gelling. The PCLS were cut with a vibratome (7000SMZ-2 Vibratome, Campden Instruments, Loughborough, England) to a thickness of 500 µm and punched to a diameter of 4 mm. Slices were cut at a speed of 10-12 µm/sec with a frequency of 90 Hz and amplitude of 1.5 mm. Punches were maintained in culture in DMEM/F12 medium supplemented with 0.1% fetal bovine serum (FBS, Gibco Life Science Technologies, Grand Island, New York, USA) and 1% by volume of 10 ,000 units/mL penicillin, 10 mg/mL streptomycin and 25 µg/mL amphotericin B antibiotic/antifungal solution (Bioreagent Sigma Aldrich). Cultured at of 37°C and 5% CO_2_ in 96 well plates with changes of medium every other day. Samples were kept for 24, 48, and 120 hours. At the appropriate time point, punches were fixed in 10% formalin (Fisher Scientific, UK) for one hour and then transferred to phosphate buffered solution. Punches then underwent processing, paraffin-embedding, and haematoxylin and eosin staining as reported for the biopsies. Bright-field images were acquired on an Olympus CKX53 microscope (Olympus Life Sciences, Tokyo, Japan) and then scored by three blinded scorers under a modified version of the biopsy scoring criteria. Scoring utilized the same scale and criteria with the exclusion of hyaline membranes, haemorrhage and atelectasis given the process by which the PCLS were produced.


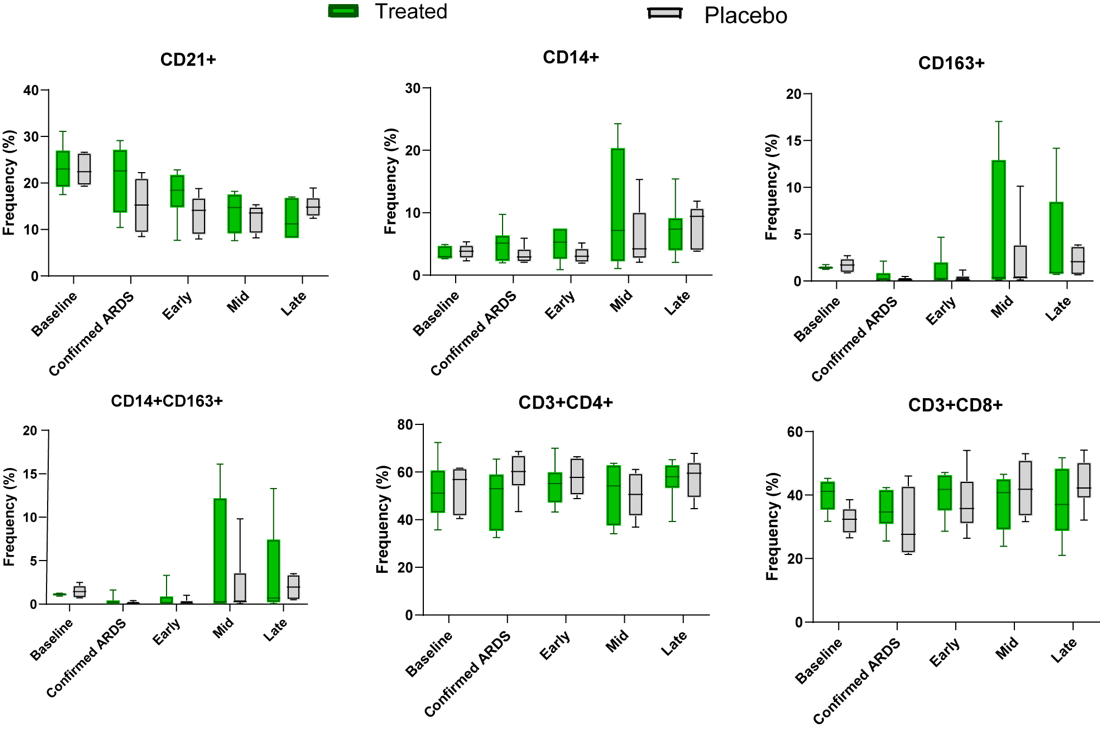


**Figure S1: Flow cytometry on isolated peripheral blood mononuclear cells (PBMCs).** Phenotypic analysis of hematopoietic cell specific surface marker profiles of PBMCs isolated for treated and placebo groups (n=6), including B cells, monocytes, macrophages and lymphocytes. Samples were analysed before administration of lipopolysaccharide (LPS), after confirmed ARDS and in the early, mid, and lates phases of the monitoring following MSC or placebo administration, respectively. Data are presented as cell frequency [%] in box and whiskers plots with min to max.


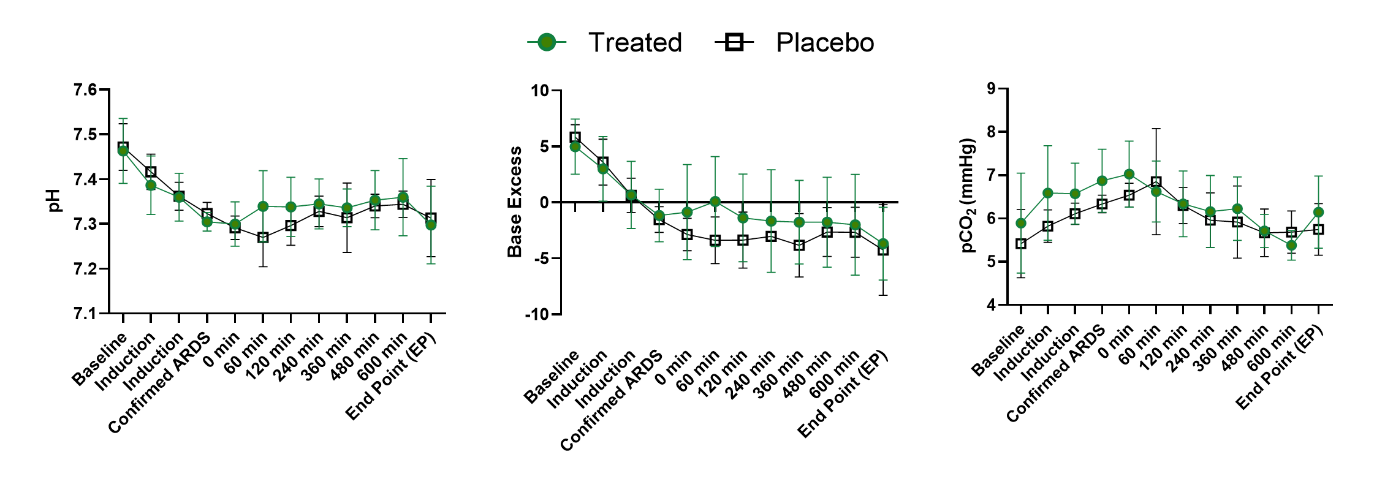
 **Figure S2: Measures of pulmonary gas exchange and lung mechanics following administration of treatment or placebo**. This included pH, base excess (mmol/L), and pCO_2_ (mmHg), the trends of which can be seen over the experimental timeline. Multiple two-sided Mann–Whitney tests were used to compare the groups when data were not normally distributed. Mixed-effects analysis with Šídá’'s multiple comparisons test was used to compare timepoints within each group. Data are presented as mean **±** standard deviation unless otherwise stated.


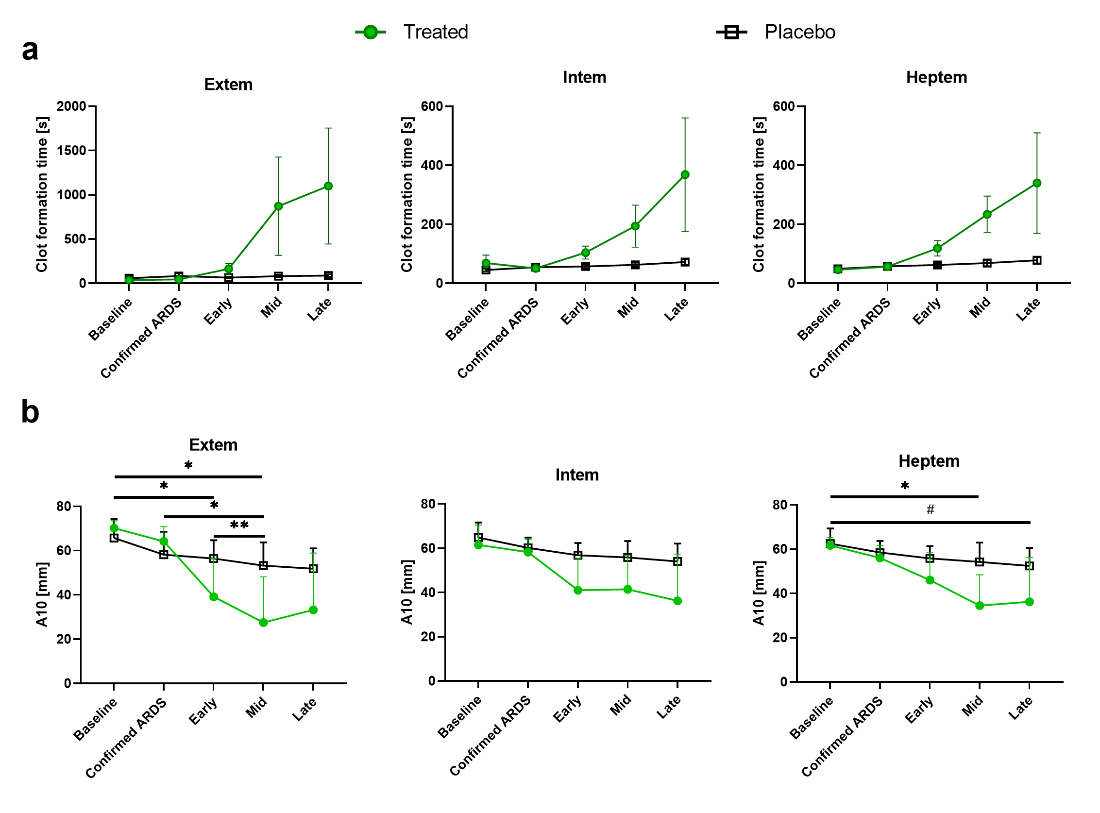


**Figure S3:** **Monitoring of haemostasis by rotational thromboelastometry (ROTEM)**. Comparison of extrinsic (EXTEM) and intrinsic (INTEM) clotting parameters, and heparin modifications (HEPTEM) throughout the experiment. Samples were analysed before administration of lipopolysaccharide, after confirmed ARDS and in the early, mid, and lates phases of the monitoring following MSC or placebo administration, respectively. **a)** Clot formation time in seconds and **b)** amplitude at 10 minutes in mm for EXTEM, INTEM, and HEPTEM were performed for the treated (green circles) and placebo (black squares) groups. Multiple two-sided Mann–Whitney tests were used to compare the groups when data were not normally distributed. Mixed-effects analysis with Šídá’'s multiple comparisons test was used to compare timepoints within each group. *p < 0.05 for comparisons within the treated group, ^#^p < 0.05 for comparisons within the placebo group. Data are presented as mean **±** standard deviation.


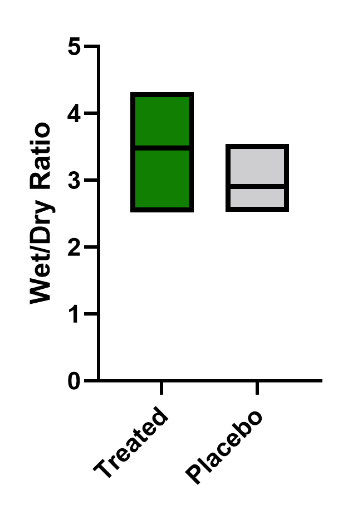


**Figure S4**: **Measure of the wet/dry ratio.** Biopsies from either treatment or placebo were taken at the end of the experimental timeframe and assessed for their wet/dry weight ratio. A Mann-Whitney test was conducted to compare the two groups, (p = 0.3939). Data are presented as box of minimum and maximum values with a bar at the mean.


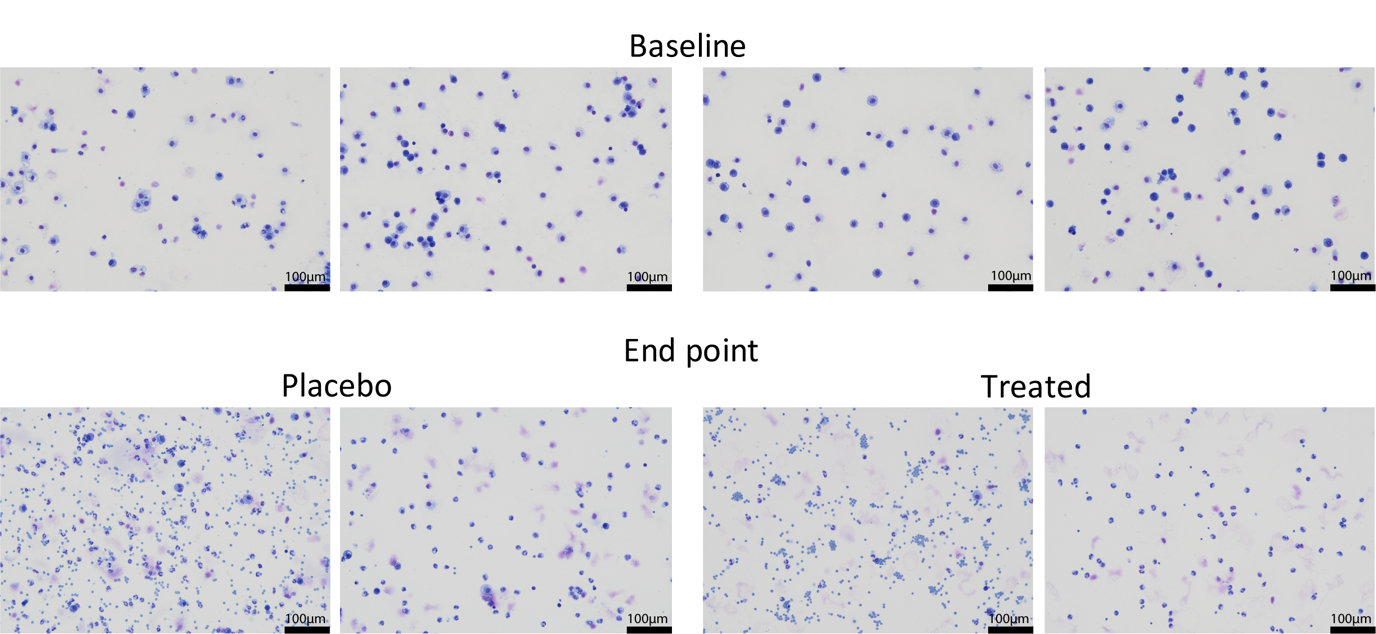
 **Figure S5:** **Representative images of bronchoalveolar lavage fluid (BALF) staining.** Cells contained in BALF were spun onto slides and stained with May-Grunwald-Giemsa stain for quantification from the treated group (n=6) and placebo group (n=6). Images were taken with 20x magnification and scale bars represent 100 µm.


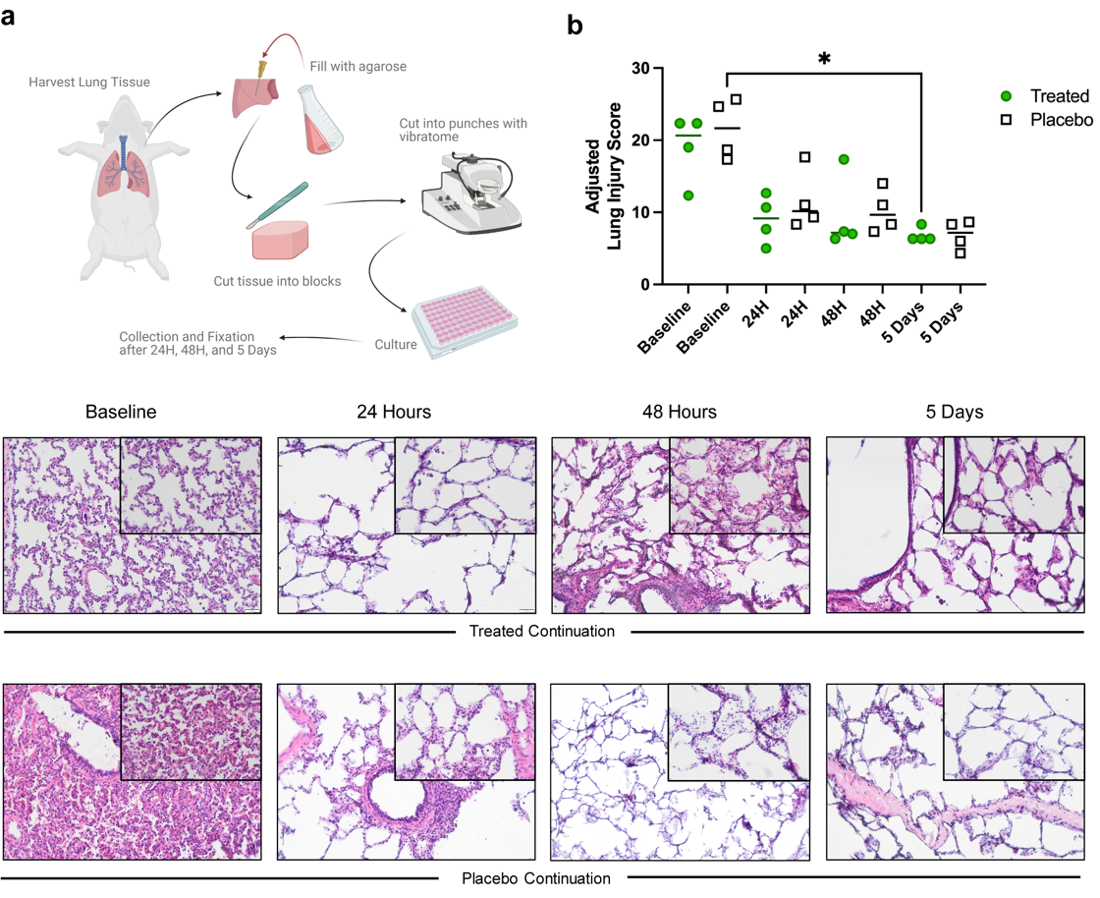


**Figure S6**: **Precision cut lung slices (PCLS) continued experimental conditions in an ex vivo setting. a)** Overview of PCLS generation. Lungs were harvested *en bloc* and then filled with agarose and cut to precise dimensions using a vibratome. They were maintained in culture for a maximum of 5 days. **c)** Blinded scoring using an adjusted lung injury score for PCLS is shown for treated subjects (n=4) and placebo subjects (n=4) at different timepoints with bars at the median value. *p < 0.0396 for comparison between the treated five day score and the placebo baseline. **c)** Representative images of haematoxylin and eosin (H&E) staining of PCLS punches during the in vitro continuation of the experiment for the treated (n=4) and placebo groups (n=4). Statistically significant differences between groups were compared with a Kruskal–Wallis test using Dunn’s multiple comparisons. *p < 0.05, Data are presented as mean ± standard deviation unless otherwise stated. Analyses were conducted on treated group (n=4) and placebo group (n=4).
